# Isolation and Description of *Catonella massiliensis* sp. nov., a Novel *Catonella* Species, Isolated from a Stable Periodontitis Subject

**DOI:** 10.3390/pathogens10030367

**Published:** 2021-03-19

**Authors:** Angéline Antezack, Manon Boxberger, Bernard La Scola, Virginie Monnet-Corti

**Affiliations:** 1 Ecole de Médecine Dentaire, Faculté des Sciences Médicales et Paramédicales, Aix-Marseille Université, 27 boulevard Jean Moulin, 13385 Marseille, France; angeline.antezack@univ-amu.fr; 2 Assistance Publique-Hôpitaux de Marseille (AP-HM), Hôpital Timone, Service de Parodontologie, 264, rue Saint Pierre, 13385 Marseille, France; 3 Institut de Recherche pour le Développement (IRD), Aix-Marseille Université, Assistance Publique-Hôpitaux de Marseille (AP-HM), MEPHI, 27 boulevard Jean Moulin, 13005 Marseille, France; manon.boxberger@hotmail.fr (M.B.); bernard.la-scola@univ-amu.fr (B.L.S.); 4 IHU Méditerranée Infection, 19–21 boulevard Jean Moulin, 13005 Marseille, France

**Keywords:** *Catonella*, gingival health, saliva, culturomics, sp. nov.

## Abstract

The genus *Catonella* currently counts a unique species, *C. morbi*, isolated from periodontal pockets and associated with periodontitis and endodontic infections. This study contributed to the taxonomical and clinical knowledge of this genus by describing a novel species isolated from a saliva sample from a man in clinical gingival health following successful treatment of periodontitis. Morphological and chemotaxonomic characteristics were investigated using different growth conditions, pH, and temperature. Cellular fatty acid methyl ester (FAME) analysis was conducted by gas chromatography/mass spectrometry (GC/MS). Phylogenetic analysis based on 16S rRNA, orthologous average nucleotide identity (OrthoANI), and digital DNA-DNA hybridization (dDDH) relatedness were performed. Strain Marseille-Q4567^T^ was found to be an anaerobic and non-spore-forming rod-shaped bacterium that grew at 28–41.5 °C (optimum 37 °C), pH 6.5–8.5 (optimum pH 7.5), and 5–10 g/L of NaCl (optimum 5 g/L). The predominant cellular fatty acid was C16:0 (64.2%), followed by unsaturated structures C18:1n9 (12.5%) and C18:2n6 (7.8%). Based on 16S rRNA sequence comparison, the closest phylogenetic neighbor was *C. morbi* ATCC 51271^T^ (98.23% similarity). The OrthoANI and dDDH values between strain Q4567^T^ and *C. morbi* ATCC 51271^T^ were respectively 79.43% and 23.8%. Therefore, we concluded that strain Marseille-Q4567^T^ represents a novel species of the genus *Catonella*, for which the name *Catonella massiliensis* sp. nov. is proposed (= CSUR Q4567).

## 1. Introduction

The human oral microbiome consists of a wide range of microorganisms, including bacteria, fungi, protozoa, viruses, and archaea and is thus, after the gut, the second largest human microbial community [1]. The symbiotic relationship between the oral microbiome and host ensures oral health [2]. The rupture of this balance and the establishment of a dysbiotic microbial community leads to oral disease, the most common being tooth decay and periodontal disease [3,4,5,6]. With the advent of next-generation sequencing and recent developments in bioinformatics, research on the diversity and the role of human oral microorganisms has increased dramatically [7]. The expanded Human Oral Microbiome Database (eHOMD) currently includes a total of 775 microbial species, of which 57% are officially named, 13% unnamed but cultivated, and 30% are known only as uncultivated phylotypes [8]. Several strategies have been used to cultivate “unculturable” bacteria, including media supplementation, diffusion chambers, and co-culture, as the isolation and culture of bacterial species are the only way to characterize them phenotypically and genotypically in an exhaustive way, and also to study their potential virulence [9,10]. The oral microbiome bacteria not yet cultured are all as likely to play a role in oral homeostasis or dysbiosis as cultured bacteria. It thus appears clear that the development of optimal culture means has a first order role in the investigation of oral microbial diversity and its potential implications in oral health or the development of dental and periodontal disease. Periodontitis is a chronic multifactorial inflammatory disease associated with dysbiotic plaque biofilms, which lead to the progressive destruction of the supporting structures of the teeth [11]. Periodontitis can be successfully treated, and stable periodontitis subjects have a bleeding on probing score in <10% of sites, shallow probing depths of 4 mm or less, and no 4 mm sites with bleeding on probing, optimal improvement in other clinical parameters, and lack of progressive periodontal destruction [12].

The genus *Catonella* (N.L. fem. dim. n. *Catonella*, in honor of Elizabeth P. Cato, an American microbiologist) belongs to the large family *Lachnospiraceae* and was first introduced into the literature by Moore and Moore (1994) [13]. *C. morbi*, isolated from periodontal pockets, is the only species of the genus *Catonella* currently described and has only been found in humans. This species was described as a putative periodontal pathogen, as it was more abundantly found in chronic periodontitis subjects [14]. Moreover, levels of *C. morbi* in subjects with chronic periodontitis decreased significantly after treatment in responsive sites, but not in refractory sites [15]. Recently, *C. morbi* has been thought to be involved in endodontic infections and was detected in 33% of the root canals associated with chronic apical periodontitis [16].

In the present study, we isolated strain Marseille-Q4567^T^ from a whole unstimulated saliva sample from a 51-year-old male in clinical gingival health following successful treatment of periodontitis. We used the cost-effective, accurate, and rapid routine identification by matrix-assisted laser desorption ionization time-of-flight (MALDI-TOF) mass spectrometry (MS) for the identification of our strain. We compared strain Marseille-Q4567^T^ to its closely related phylogenetic neighbors and proposed that strain Marseille-Q4567^T^ represents a novel species of the genus *Catonella*, for which the name *Catonella massiliensis* sp. nov. is proposed (=CSUR Q4567).

## 2. Results

### 2.1. Strain Isolation and Phenotypic Characteristics

**Figure 1:**
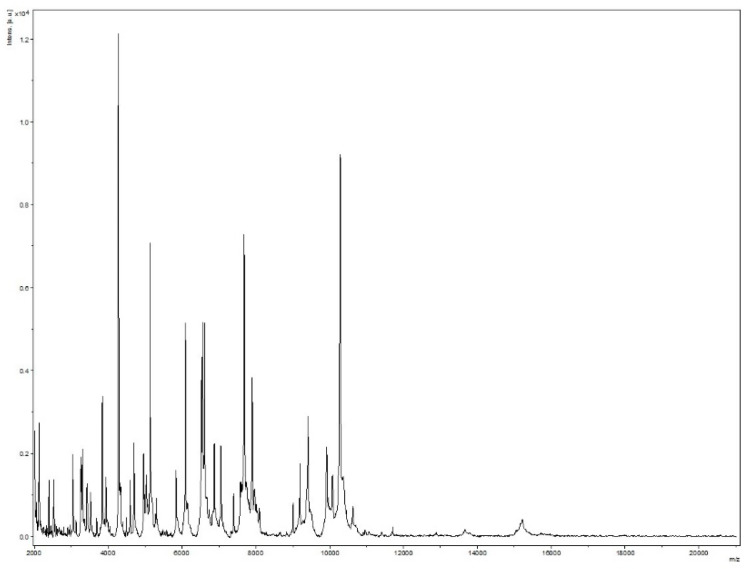
MALDI-TOF MS reference mass spectrum for strain Marseille-Q4567^T^. Spectra from 12 individual colonies were compared and a reference spectrum was generated.

Strain Marseille-Q4567^T^ was isolated from a whole unstimulated saliva sample provided by a 51-year-old male in clinical gingival health following successful treatment of periodontitis, and living in Marseille, France. Strain Marseille-Q4567^T^ could not be identified by MALDI-TOF MS, as the score was lower than 1.8 (Figure 1).

**Figure 2:**
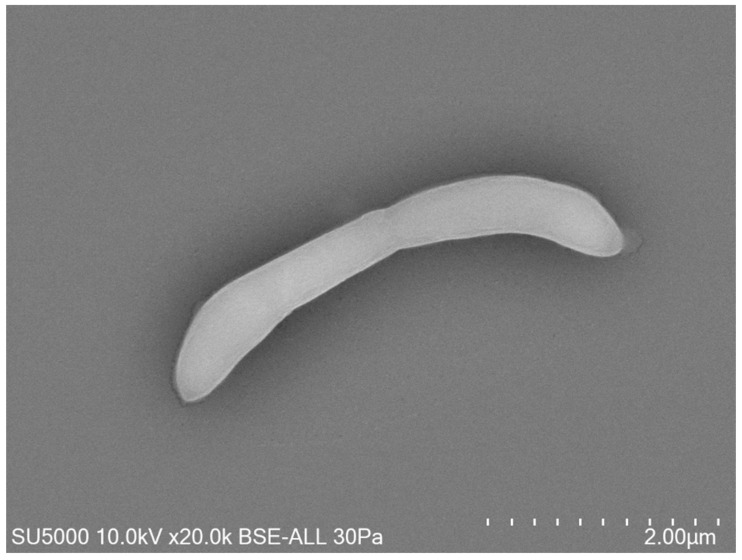
Micrograph electron microscopy of strain Marseille-Q4567^T^. Scales and acquisition settings are shown in the figure.

Growth was observed on Columbia agar with 5% sheep blood (BioMérieux, Marcy-l’Etoile, France) at 37 °C after 24 h of incubation in an anaerobic atmosphere. Growth was also achieved in microaerophilic but not in aerobic conditions. The temperature range of the strain was determined to be 28–41.5 °C, with an optimum growth temperature 37 °C. The bacterial cells tolerated a pH of 6.5 to 8.5 (optimum pH 7.5) and an NaCl content ranging from 5 to 10 g/L (optimum 5 g/L). After 10 min of thermal shock at 80 °C, this bacterium did not grow at 37 °C on Columbia agar with 5% sheep blood, which indicated the absence of spore formation. Surface colonies on Columbia agar with 5% sheep blood (BioMérieux, Marcy-l’Etoile, France) incubated at 37 °C for 2 days were approximately between 0.1 and 0.5 mm in diameter, smooth, circular, and translucent. Bacterial cells were Gram-negative curved rods, round-tipped, and approximatively 0.3 to 0.5 μm wide and 3.9 to 4.1 μm long, as determined by field emission scanning electron microscopy (Figure 2).

Using an API 50 CH strip, positive reactions were obtained for glycerol, erythritol, D-arabinose, L-arabinose, D-ribose, D-xylose, L-xylose, D-adonitol, methyl βD-xylopyranoside, D-galactose, D-glucose, D-fructose, D-mannose, L-sorbose, L-rhamnose, dulcitol, inositol, D-mannitol, D-sorbitol, methyl αD-mannopyranoside, Methyl αD-glucopyranoside, N-acetyl-glucosamine, amygdalin, arbutin, esculin ferric citrate, salicin, D-cellobiose, D-maltose, D-lactose, D-melibiose, D-saccharose, D-trehalose, Inulin, D-melezitose, D-raffinose, amidon, glycogen, xylitol, gentiobiose, D-turanose, D-lyxose, D-tagatose, D-fucose, L-fucose, D-arabitol, L-arabitol, potassium gluconate, potassium 2-ketogluconate, and potassium 5-ketogluconate. Using an API ZYM strip, a positive result was shown for alkaline phosphatase, leucine arylamidase, acid phosphatase, naphthol-AS-BI-phosphohydrolase, α-galactosidase, β-galactosidase, α-glucosidase, β-glucosidase, and N-acetyl-β-glucosaminidase. Strain Marseille Q4567^T^ was negative for oxidase and catalase activities. Comparison of phenotypic characteristics between strain Marseille-Q4567^T^ and its closely related species—*Cuneatibacter caecimuris, Faecalicatena orotica*, *Faecalicatena contorta*, *Faecalicatena fissicatena*, *Anaerocolumna cellulosilytica*, *Catonella morbi*, and *Herbinix luporum*—is listed in Table 1.

The most abundant fatty acid was C_16:0_ (64.2%), followed by unsaturated structures C_18:1n9_ (12.5%) and C_18:2n6_ (7.8%). No branched fatty acids were described. Minor amounts of other unsaturated and saturated structures were also detected (Table 2).

### 2.2. Genome Sequencing Information and Genome Properties

**Figure 3:**
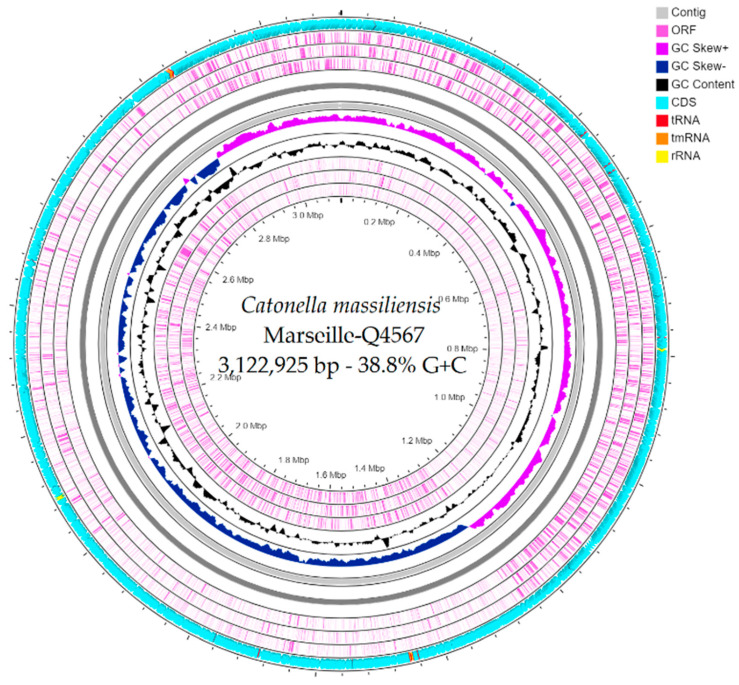
A circular map generated using the CGView Server^BETA^ [21] showing a complete view of the genome of strain Marseille-Q45657^T^.

The genome size of strain Marseille-Q4567^T^ was 3,122,925 bp long with a 38.8% G+C content. It was assembled into 3 contigs with a mean coverage of 131.6. It was deposited into GenBank under the accession number JAEPRJ000000000. Of the 2805 predicted genes, 2747 were protein-coding genes and 58 were RNAs (four 5S rRNA, four 16S rRNA, four 23S rRNA, 42 tRNAs, and 4 ncRNA). Genes with putative function (by COGs) were 1628 for strain Marseille-Q4567^T^ (Table 3). Finally, 1396 genes (49%) were annotated as hypothetical proteins for strain Marseille-Q4567^T^. A circular map showing a complete view of the genome of strain Marseille-Q4567^T^ is shown in Figure 3. The mass screening for antimicrobial and virulence genes revealed the presence of lsa(C)_1 gene (92.02% identity, 100.00% coverage) which confers resistance to lincomycin, clindamycin, dalfopristin, pristinamycin_IIA, virginiamycin_M, and tiamulin.

### 2.3. Comparison with Closely Related Bacterial Strains

**Figure 4:**
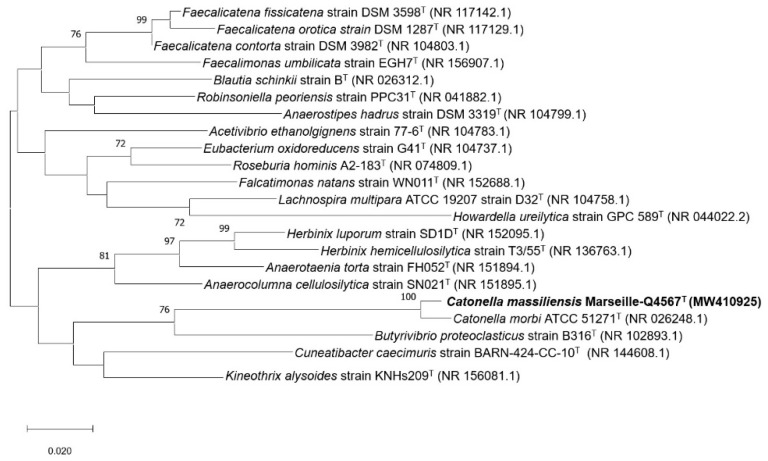
Maximum likelihood tree based on the comparison of 16S rRNA gene sequences showing the phylogenetic relationships of *Catonella massiliensis* Marseille-Q4567^T^ and other closely related species. Bootstrap values (expressed as percentages of 1000 replications) are displayed at the nodes. Only bootstrap values of 70% or greater are shown. Type strains are indicated with superscript T. GenBank accession numbers of 16S rRNA indicated in parentheses. Sequences were aligned using MUSCLE with default parameters, phylogenetic inference was obtained using the Maximum likelihood method and MEGA X software [22]. Bootstrap values obtained by repeating the analysis 1000 times to generate a majority consensus tree are indicated at the nodes. There was a total of 1392 positions in the final dataset.

16S rDNA-based similarity analysis of strain Marseille-Q4567^T^ against GenBank yielded the highest nucleotide sequence similarities of 98.23% sequence identity with *C. morbi* ATCC 51271^T^ (GenBank accession no. NR_026248.1). The 16S rRNA gene sequence was deposited into GenBank under the accession number MW410925. The phylogenetic tree highlighting the position of the strain relative to other closely related species is shown in Figure 4.

The genome of strain Marseille-Q4567^T^ was compared to the available genomes of nine closely related bacterial strains. The genome size of our strain (3,122,925 bp) was larger than that of *H. luporum* and *E. oxidoreducens* but smaller than that of *C. caecimuris*, *C. morbi*, *R. hominis*, *F. fissicatena*, *A. cellulosilytica*, *F. contorta*, and *F. orotica* (Table 4). The G+C content of strain Marseille Q4567^T^ (38.8%) was smaller than that of all compared species except *H. luporum* (35.3%), *A. cellulosilytica* (36.7%), and *C. morbi* (37.1%).

**Figure 5:**
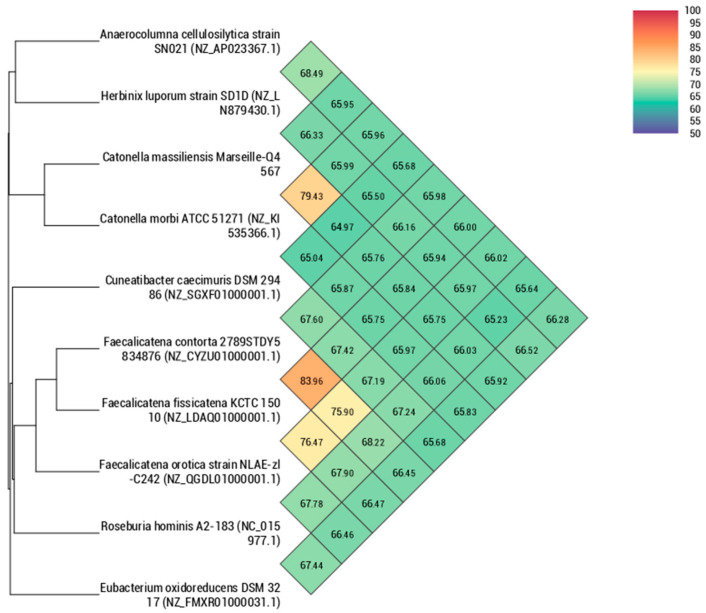
Heatmap generated with orthologous average nucleotide identity (OrthoANI) values calculated using the OAT software for strain Marseille-Q4567^T^ with other closely related species validly described.

Using dDDH analysis, strain Marseille-Q4567^T^ exhibited values ranging from 22.9% [20.6–25.3%] with *H. luporum* to 28.4% [26.1–30.9%] with *C. caecimuris* (Table 5). These values were lower than the 70% threshold used for delineating prokaryotic species, thus confirming that strain Marseille-Q4567^T^ represents a new species [10]. In addition, using OrthoANI analysis, strain Marseille-Q4567^T^ exhibited values ranging from 64.97% with *C. caecimuris* to 79.43% with *C. morbi* (Figure 5).

**Figure 6:**
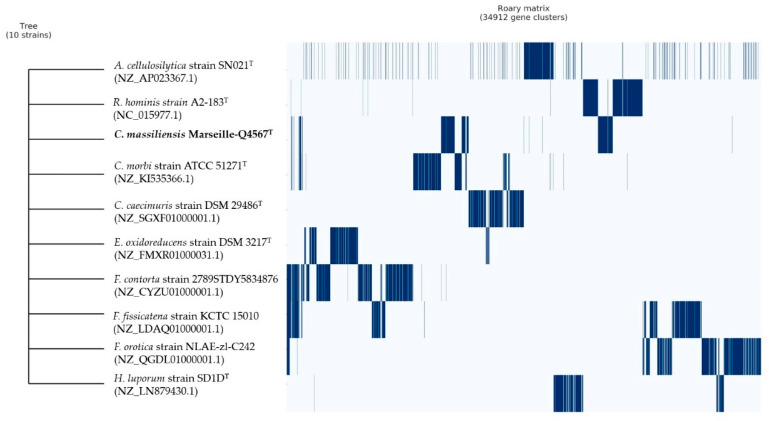
Pangenome analysis strain of Marseille-Q4567^T^ whole-genome sequences. A maximum likelihood tree was constructed from the accessory genome elements (left). The presence (blue) and the absence (white) of accessory genome elements are presented on the right.

Finally, pangenome analysis of strain Marseille-Q4567^T^ showed a total of 34,912 gene clusters distributed as follows: (Core genes = 0), (soft core genes = 0), (shell genes = 1171), and (cloud genes = 33,741), respectively (Figure 6).

### 2.4. Description of Catonella Massiliensis *sp. nov.*

*Catonella massiliensis* (mas.si.li.en’sis L. masc./fem. adj. massiliensis, pertaining to Massilia, the ancient Roman name of Marseille, France, where the organism was isolated).

Cells were Gram-negative, anaerobic, curved rods, round-tipped, and approximatively 0.3 to 0.5 μm wide and 3.9 to 4.1 μm long. Colonies on Columbia agar with 5% sheep blood (BioMérieux, Marcy-l’Etoile, France) incubated at 37 °C for 2 days were approximatively 0.1 to 0.5 mm in diameter, smooth, circular, and translucent. The temperature range for growth was 28–41.5 °C (optimum 37 °C). The cells grew at pH 6.5 to 8.5 (optimum 7.5) and with an NaCl content ranging from 5 to 10 g/L (optimum 5 g/L). Test results were positive for the fermentation of glycerol, erythritol, D-arabinose, L-arabinose, D-ribose, D-xylose, L-xylose, D-adonitol, methyl βD-xylopyranoside, D-galactose, D-glucose, D-fructose, D-mannose, L-sorbose, L-rhamnose, dulcitol, inositol, D-mannitol, D-sorbitol, methyl αD-mannopyranoside, Methyl αD-glucopyranoside, N-acetyl-glucosamine, amygdalin, arbutin, esculin ferric citrate, salicin, D-cellobiose, D-maltose, D-lactose, D-melibiose, D-saccharose, D-trehalose, Inulin, D-melezitose, D-raffinose, amidon, glycogen, xylitol, gentiobiose, D-turanose, D-lyxose, D-tagatose, D-fucose, L-fucose, D-arabitol, L-arabitol, potassium gluconate, potassium 2-ketogluconate, and potassium 5-ketogluconate. According to the API ZYM system, cells were positive for alkaline phosphatase, leucine arylamidase, acid phosphatase, naphthol-AS-BI-phosphohydrolase, α-galactosidase, β-galactosidase, α-glucosidase, β-glucosidase, and N-acetyl-β-glucosaminidase. The predominant cellular fatty acid was C_16:0_ (64.2%), followed by unsaturated structures C_18:1n9_ (12.5%) and C_18:2n6_ (7.8%). The genome size of strain Marseille-Q4567^T^ was 3,122,925 bp long with a 38.8% G+C content. The type of strain, Marseille-Q4567^T^ (CSUR Q4567), was isolated from a sample of unstimulated whole saliva from a male with stable periodontitis. The sequence data of the 16S rRNA gene of Marseille-Q4567^T^ and the whole genome have been deposited in the GenBank database under accession numbers MW410925 and JAEPRJ000000000, respectively.

## 3. Discussion

The human oral microbiome is a complex ecosystem whose balance with the host immune system is necessary in maintaining oral health. In this study, we aimed at isolating and describing a novel bacterial species using culturomics and taxonogenomic strategies in order to contribute new knowledge on the diversity of the oral microbiome.

The phenotypic and phylogenetic analysis of our strain Marseille-Q4567^T^ revealed several different characteristics when compared to other members of the family *Lachnospiraceae*, suggesting that it could be classified as a new species. The family *Lachnospiraceae* is a large family currently composed of 57 genera with a validly published and correct name. Among the members of the family *Lachnospiraceae*, strain Marseille-Q4567^T^ shared the highest 16S rRNA gene sequence similarities (98.23%) with *C. morbi* ATCC 51271^T^, an anaerobic Gram-negative bacilli isolated from diseased periodontal pockets [13]. *C. morbi* has been thought to be involved in periodontal and endodontic infections [14,16]. Here, our strain Marseille-Q4567^T^ was isolated from the saliva of a 51-year-old male in clinical gingival health following successful treatment of periodontitis. The discovery of this new species alone does not allow us to associate its presence with the patient’s periodontal condition. In this aim, additional studies exploring the prevalence of our strain *Catonella massiliensis* Marseille-Q4567^T^ according to the presence or absence of periodontal diseases will be of great interest. In addition, as stable periodontitis patients remain at higher risk for recurrent disease, it would be interesting to monitor the presence of *Catonella* species in treated and maintained periodontitis subjects and to correlate it with potential recurrence of the disease. Further studies will also be needed to investigate the potential involvement of the genus *Catonella* in gingival inflammation and periodontal destruction.

The genomic content (dDDH, orthoANI, and pangenome) with biochemical characteristics clearly indicated that strain Marseille-Q4567^T^ could be differentiated from the closely related species *C. morbi, C. caecimuris, R. hominis, F. fissicatena, A. cellulosilytica, F. contorta, H. luporum, F. orotica*, and *E. oxidoreducens*. Based on the results from phenotypic, chemotaxonomic, genomic, and phylogenetic analyses and data, we concluded that strain Marseille-Q4567^T^ represents a novel species of the genus *Catonella*, for which the name *Catonella massiliensis* sp. nov. is proposed (=CSUR Q4567).

## 4. Materials and Methods

### 4.1. Strain Isolation and Phenotypic Tests

A sample of unstimulated whole saliva was collected from a 51-year-old male in clinical gingival health following successful treatment of periodontitis and currently referred for periodontal maintenance at the periodontal department of the Pavillon Odontologique de la Timone, Marseille, France. The patient provided signed informed consent, and the study was approved by the Comité de Protection des Personnes (C.P.P.) Sud-Ouest et Outre-Mer 1 (no. ID RCB: 2020-A01234-35–CPP 1-20-075 ID 9806). Briefly, a 2-mL volume of whole unstimulated saliva was collected into a 50-mL centrifuge tube and transported to an anaerobic workstation within 1 h. After vortexing for 30 s, a 10-fold dilution series of the sample was prepared in phosphate-buffered saline 1x. Columbia agar sheep blood plates (BioMérieux, Marcy l’Etoile, France) were inoculated with 50 μL each of a 10^−4^ to 10^−8^ diluted plaque suspension. After 48 h of incubation in an anaerobic atmosphere (AnaeroGen Compact; Oxoid, Thermo Scientific, Dardilly, France) at 37 °C, the culture plates were inspected using a magnifying glass, and any microcolonies or colonies showing satellitism were passaged onto a fresh Columbia agar sheep blood plate. Matrix-assisted laser desorption/ionization time-of-flight (MALDI-TOF) mass spectrometry (MS) protein analysis was performed with a Microflex LT mass spectrometer (Bruker Daltonics, Bremen, Germany; external mass spectrometer calibration accuracy ± 300 ppm), as previously reported [23]. Briefly, each isolate colony was deposited on a 96 polished steel MALDI target and then coated with 1 μL of matrix solution containing α-cyano-4-hydroxycinnamic acid diluted in 500 μL of acetonitrile, 250 μL of 10% trifluoroacetic acid, and 250 μL of HPLC-grade water. The matrix sample was then crystallized by air-drying at room temperature as previously described [24]. Two spots were systematically created for each colony, and each isolate was characterized by at least 12 spots. The obtained spectra were imported into the BioTyper-RTC^TM^ version 3.0 software (Bruker Daltonics GmbH) and analyzed by standard pattern matching (with default parameter settings). Interpretation of the scores was carried out as previously reported [23]. One purified strain, designated Marseille-Q4567^T^ and deposited in the Collection de Souches de l’Unité des Rickettsies under accession number Q4567, could not be identified by MALDI-TOF MS.

Gram staining was carried out using standard Gram stain, and morphological characteristics were observed with a field emission scanning electron microscope (SU5000 FE-SEM, Hitachi High-Tech, HHT, Tokyo, Japan) using cultures grown on Columbia agar with 5% sheep blood (BioMérieux, Marcy l’Etoile, France) at 37 °C for 24 h. A colony was collected from the agar and immersed in a 2.5% glutaraldehyde fixative solution. The slide was gently washed in water, air-dried, and examined with a SU5000 (Hitachi SU5000) operated at 10.0 kV. Subculture of the strain Marseille-Q4567^T^ was attempted at a wide range of temperatures (25, 28, 31.5, 37, 41.5, and 56 °C) on Columbia agar with 5% sheep blood and in different conditions of pH (5.5, 6.5, 7.5, and 8.5) and salinity (5, 10, and 15g/L) on Columbia agar bases (BioMérieux, Marcy l’Etoile, France). The growth of the strain was also tested under anaerobic (AnaeroGen Compact; Oxoid, Thermo Scientific, Dardilly, France), microaerophilic (campyGEN; Oxoid, Thermo Scientific, Dardilly, France), and aerobic conditions at 37 °C for 48 h. API ZYM and API 50 CH kits (BioMérieux, Marcy l’Etoile, France) were used to perform biochemical analysis in accordance with the manufacturer’s instructions. Oxidase (MASTDISCS^®^ ID, Mast Group Ltd., Bootle, Merseyside, UK) and catalase (BioMérieux, Marcy l’Etoile, France) assays were also performed. The ability of strain Marseille-Q4567^T^ to form spores was evaluated following thermal shock at 80 °C for 10 min. Cellular fatty acid methyl ester (FAME) analysis was performed by GC/MS. Two samples were prepared with approximately 3 mg of bacterial biomass per tube harvested from several culture plates. Fatty acid methyl esters were prepared as described by Sasser [25]. GC/MS analyses were carried out as previously described [26]. Briefly, fatty acid methyl esters were separated using an Elite 5-MS column and monitored by mass spectrometry (Clarus 500–SQ 8 S, Perkin Elmer, Courtaboeuf, France). A spectral database search was performed using MS Search 2.0, operated with the Standard Reference Database 1A (NIST, Gaithersburg, MD, USA) and the FAMEs mass spectral database (Wiley, Chichester, UK).

### 4.2. Extraction and Genome Sequencing

The genomic DNA (gDNA) of strain Marseille-Q4567^T^ was extracted in two steps: Mechanical treatment was first performed by acid washed glass beads (G4649-500g Sigma) using a FastPrep-24™ 5G Grinder (mpBio) at maximum speed (6.5) for 90 s. Then, after 30 min lysozyme incubation at 37 °C, DNA was extracted on the EZ1 biorobot (Qiagen) with an EZ1 DNA tissues kit. The elution volume was 50 µL. The gDNA of strain Marseille-Q4567^T^ was quantified by a Qubit assay with the high sensitivity kit (Life technologies, Carlsbad, CA, USA) to 0.2 ng/µL. Genomic DNA was next sequenced on MiSeq Technology (Illumina Inc, San Diego, CA, USA) with the paired-end strategy, and was barcoded in order to be mixed respectively with 20 other genomic projects prepared with the Nextera XT DNA sample prep kit (Illumina). To prepare the paired-end library, dilution was performed to require 1 ng of each genome as input to prepare the paired end library. The “tagmentation” step fragmented and tagged the DNA. Limited cycle PCR amplification (12 cycles) completed the tag adapters and introduced dual-index barcodes. After purification on AMPure XP beads (Beckman Coulter Inc, Fullerton, CA, USA), the libraries were then normalized on specific beads according to the Nextera XT protocol (Illumina). Normalized libraries were pooled into a single library for sequencing on the MiSeq. The pooled single strand library was loaded onto the reagent cartridge and then onto the instrument along with the flow cell. Automated cluster generation and paired end sequencing with dual index reads were performed in a single 39-h run in 2x250-bp. Information totaling 5.54 Gb was obtained from a 578 K/mm^2^ cluster density with a cluster passing quality control filters of 95.5%. Within this run, the index representation for strain Marseille-Q4567^T^ was determined to index 5.97%. The 11,232,685 paired-end reads were filtered according to the read qualities.

In order to improve the genome sequence, an Oxford Nanopore approach was performed on 1D genomic DNA sequencing for the MinIon device using the SQK-LSK109 kit. A library was constructed from 1 µg genomic DNA without fragmentation or end repair. Adapters were ligated to both ends of genomic DNA. After purification on AMPure XP beads (Beckman Coulter Inc, Fullerton, CA, USA), the library was quantified by a Qubit assay with the high sensitivity kit (Life technologies, Carlsbad, CA, USA). In total, 1500 active pores were detected for the sequencing and the “What’s In My Pot (WIMP)” workflow was chosen for bioinformatic analysis in real time. After 21 h as the run time and end life of the flow cell, 177,380 reads as raw data were generated.

### 4.3. Assembly and Annotation of the Genome Sequence

The assembly was performed with a pipeline incorporating different software (Velvet [27], Spades [28], Soap Denovo [29]), and trimmed data (MiSeq and Trimmomatic [30] software) or untrimmed data (only MiSeq software). GapCloser was used to reduce assembly gaps. Scaffolds < 800 bp and scaffolds with a depth value < 25% of the mean depth were removed. The best assembly was selected using different criteria (number of scaffolds, N50, number of N).

Prokka (Galaxy v 1.14.5) was used for prediction in the open reading frame (ORF) with the default settings [31]. Deviations in the sequencing regions predicted by ORFs were excluded. BlastP was used to predict the bacterial proteome (E value of 1e03, coverage of 70% and percent identity of 30%) according to the Orthological Group (COG) database. In the absence of a match, the search for BlastP in the database [32] was extended with an E value of 1e03, coverage of 70%, and percent identity of 30%. If the length of the sequence was less than 80 amino acids (aa), an E value of 1e05 was used. The rRNA and tRNA genes were retrieved using the Prokka (Galaxy v 1.14.5) [33,34]. CGView Server^BETA^ [21] was used to generate a circular map showing a complete view of the genome of strain Marseille-Q4567^T^. Finally, a mass screening for antimicrobial and virulence genes was performed using ABRIcate (Galaxy Version 1.0.1) [35].

### 4.4. Phylogenetic Analysis and Genome Comparison

The 16S rRNA gene sequence of strain Marseille-Q4567^T^ (1532 bp) was obtained and compared with the most closely related species retrieved using NCBI BLAST (National Center for Biotechnology Information, Basic Local Alignment Search Tool; https://blast.ncbi.nlm.nih.gov/Blast.cgi, accessed on 10 January 2021) and then submitted to the GenBank database. Phylogenetic analyses were performed using MEGA X software [22], with genetic distances determined according to the Kimura two-parameter model [36] and phylogenies reconstructed with the maximum-likelihood method. The topology of the phylogenetic tree was conducted using the bootstrap method with 1000 repetitions. All positions containing gaps and missing data were eliminated from the dataset (complete deletion option). Digital DNA–DNA hybridization (dDDH) values between strain Marseille-Q4567^T^ and other closely related species were assessed using the Genome-to-Genome Distance Calculator 2.1 (GGDC) web server (http://ggdc.dsmz.de, accessed on 10 January 2021) [37]. In addition, the orthologous average nucleotide identity was calculated using OrthoANI v 0.93.1 software [38]. Finally, the pangenome distribution of strain Marseille Q4567^T^ and other closely related species was evaluated using Roary software (Galaxy v 3.13.0) [39].

## Figures and Tables

**Table 1:** Phenotypic characterization of *Catonella massiliensis* sp. nov., compared to other closely related species. +, Positive; w, weak; -, negative; ND, no data available. Strains: 1, Marseille-Q4567^T^; 2, *Cuneatibacter caecimuris* DSM 29486^T^ [17]; 3, *Faecalicatena orotica* JCM 1429^T^ [18]; 4, *Faecalicatena contorta* JCM 6483^T^ [18]; 5, *Faecalicatena fissicatena* JCM 31501^T^ [18]; 6, *Anaerocolumna cellulosilytica* strain SN021^T^ [19]; 7, *Catonella morbi* ATCC 51271^T^ [13]; 8, *Herbinix luporum* strain SD1D^T^ [20].

Characteristic	1	2	3	4	5	6	7	8
Gram stain	-	+	+	+	+	+	-	+
Spore formation	-	-	+	-	-	+	-	-
Temperature (°C)	28–41.5	37	20–40	20–40	20–40	15–40	37	40–65
pH	6.5–8.5	7.0	6.0–8.0	6.0–8.0	6.0–8.0	6.2–8.5	5.0–5.6	6.5–8.5
Catalase activity	-	ND	ND	ND	-	-	-	-
Oxidase activity	-	ND	ND	ND	ND	-	ND	ND
Acid produced from:								
L-Arabinose	+	+	+	+	+	+	-	+
Cellobiose	+	-	+	+	-	+	+	+
Lactose	+	-	+	+	-	+	+	-
Maltose	+	-	+	+	+	+	+	ND
D-Mannitol	+	-	+	-	-	-	-	ND
D-Mannose	+	-	+	+	+	+	-	+
Raffinose	+	-	+	+	-	-	+	ND
L-Rhamnose	+	+	+	+	+	-	+	ND
D-Sorbitol	+	-	+	-	-	-	-	-
Enzyme activity:								
Alkaline phosphatase	+	-	-	-	-	ND	ND	ND
C_4_ esterase	-	ND	w	w	w	ND	ND	ND
C_8_ esterase lipase	-	ND	w	w	-	ND	ND	ND
C_14_ lipase	-	ND	-	-	-	-	ND	ND
Leucine arylamidase	+	-	-	-	-	ND	ND	ND
Valine arylamidase	-	ND	-	-	-	ND	ND	ND
Cystine arylamidase	-	ND	-	-	-	ND	ND	ND
Trypsin	-	ND	-	-	-	ND	ND	ND
α-Chymotrypsin	-	ND	-	-	-	ND	ND	ND
Acid phosphatase	+	ND	+	+	w	ND	ND	ND
Naphthol-AS-BI-phosphohydrolase	+	ND	w	w	w	ND	ND	ND
α-Galactosidase	+	+	+	+	+	ND	ND	ND
β-Galactosidase	+	+	+	+	-	ND	ND	ND
β-Glucuronidase	-	+	+	-	-	ND	ND	ND
α-Glucosidase	+	+	+	w	+	ND	ND	ND
β-Glucosidase	+	+	+	-	-	ND	ND	ND
N-Acetyl-β-glucosaminidase	+	-	-	-	-	ND	ND	ND
α-Mannosidase	-	ND	-	-	-	ND	ND	ND
α-Fucosidase	-	-	+	-	-	ND	ND	ND

**Table 2:** Cellular fatty acid compositions of strain Marseille-Q4567^T^ and its closely related species. DMA, dimethyl acetal; ALDE, aldehyde. -, not detected; TR, trace amounts (<1%); ND, no data available. Strains: 1, Marseille-Q4567^T^; 2, *Cuneatibacter caecimuris* DSM 29486^T^ [17]; 3, *Faecalicatena orotica* JCM 1429^T^ [18]; 4, *Faecalicatena contorta* JCM 6483^T^ [18]; 5, *Faecalicatena fissicatena* JCM 31501^T^ [18]; 6, *Anaerocolumna cellulosilytica* strain SN021^T^ [19]; 7, *Catonella morbi* ATCC 51271^T^ [13]; 8, *Herbinix luporum* strain SD1D^T^ [20].

Fatty Acid	1	2	3	4	5	6	7	8
C_12:0_	TR	ND	1.6	3.3	2.6	ND	ND	ND
C_13:0_	TR	ND	-	-	-	ND	ND	ND
C_14:0_	6.4	6.2	6.0	9.3	9.2	0.6	42	14.0
C_14:0_ DMA	ND	ND	-	-	-	1.5	14	9.1
C_15:0_	TR	ND	-	-	-	ND	ND	ND
C_16:0_	64.2	43.4	12.0	15.6	15.6	5.5	12	19.9
C_16:0_ ALDE	-	ND	-	-	-	20.6	ND	2.9
C_16:0_ DMA	ND	ND	2.0	3.4	3.4	17.4	ND	5.3
C_16:0_ 3-OH	-	ND	ND	ND	ND	1.2	ND	ND
C_16:1ω9c_ DMA	ND	ND	ND	ND	ND	3.8	ND	ND
C_16:1n9_	-	ND	ND	ND	ND	ND	2	1.2
C_17:0_	TR	ND	-	-	-	ND	ND	ND
C_17:1 ω8c_	ND	8.3	ND	ND	ND	ND	ND	ND
C_18:0_	6.6	ND	1.4	-	3.9	ND	4	1.2
C_18:1n7_	1.3	ND	ND	ND	ND	ND	ND	ND
C_18:1n9_	12.5	ND	25.9	21.9	22.9	ND	5	ND
C_18:1ω9C_ DMA	ND	ND	37.0	36.3	28.6	3.1	ND	ND
C_18:1ω7C_ DMA	ND	ND	2.4	-	4.0	9.0	ND	ND
C_18:2n6_	7.8	ND	ND	ND	ND	ND	ND	ND
C_19:0_ cyclo 9,10 DMA	ND	ND	ND	ND	ND	ND	ND	38.3
C_19:0_ cyclo 11,12 DMA	ND	ND	ND	ND	ND	1.4	ND	ND

**Table 3:** Number of genes associated with the general clusters of orthologous group (COG) functional categories of strain Marseille-Q4567^T^.

*Code*	*Strain Marseille-Q4567 ^T^*	*Description*
*[J]*	145	Translation, ribosomal structure, and biogenesis
*[A]*	0	RNA processing and modification
*[K]*	150	Transcription
*[L]*	137	Replication, recombination, and repair
*[B]*	0	Chromatin structure and dynamics
*[D]*	29	Cell cycle control, cell division, chromosome partitioning
*[Y]*	0	Nuclear structure
*[V]*	76	Defense mechanisms
*[T]*	82	Signal transduction mechanisms
*[M]*	64	Cell wall/membrane/envelope biogenesis
*[N]*	49	Cell motility
*[Z]*	0	Cytoskeleton
*[W]*	0	Extracellular structures
*[U]*	38	Intracellular trafficking, secretion, and vesicular transport
*[O]*	60	Posttranslational modification, protein turnover, chaperones
*[X]*	0	Mobilome: Prophages, transposons
*[C]*	74	Energy production and conversion
*[G]*	183	Carbohydrate transport and metabolism
*[E]*	141	Amino acid transport and metabolism
*[F]*	51	Nucleotide transport and metabolism
*[H]*	31	Coenzyme transport and metabolism
*[I]*	47	Lipid transport and metabolism
*[P]*	86	Inorganic ion transport and metabolism
*[Q]*	21	Secondary metabolites biosynthesis, transport, and catabolism
*[R]*	214	General function prediction only
*[S]*	134	Function unknown

**Table 4:** Genomic comparison (sequence size, number of contigs, G+C contents, and gene content) between strain Marseille-Q4567^T^ and nine closely related bacterial strains.

Species	Genome Size (bp)	Number of Contigs	G+C Content (%)	Gene Content
*Catonella massiliensis* Marseille-Q4567^T^	3,122,925	3	38.8	2849
*Cuneatibacter caecimuris* DSM 29486^T^ (NZ_SGXF01000001.1)	3,462,725	16	49.1	3268
*Eubacterium oxidoreducens* DSM 3217^T^ (NZ_FMXR01000031.1)	2,912,287	33	39.8	2700
*Faecalicatena orotica* strain NLAE-zl-C242 (NZ_QGDL01000001.1)	5,717,637	33	44.7	5174
*Roseburia hominis* A2-183^T^ (NC_015977.1)	3,592,125	1	48.5	3349
*Faecalicatena contorta* 2789STDY5834876 (NZ_CYZU01000001.1)	5,545,490	139	45.9	5148
*Faecalicatena fissicatena* KCTC 15010 (NZ_LDAQ01000001.1)	5,014,239	184	45.6	4411
*Anaerocolumna cellulosilytica* strain SN021^T^ (NZ_AP023367.1)	5,430,627	1	36.7	4545
*Catonella morbi* ATCC 51271^T^ (NZ_KI535366.1)	3,479,204	8	37.1	3155
*Herbinix luporum* strain SD1D^T^ (NZ_LN879430.1)	2,609,352	1	35.3	2430

**Table 5:** Numerical DNA–DNA hybridization values (%) obtained by comparison between strain Marseille-Q4567^T^ and other closely related species using GGDC formula 2 software (DDH estimates based on HSP identities/length) [https://ggdc.dsmz.de/ggdc.php#, accessed on 10 January 2021]. Type strains are indicated with superscript T.

Species	1	2	3	4	5	6	7	8	9	10
1 *Catonella massiliensis* Marseille-Q4567^T^	100.00	28.4 [26.1–30.9]	27.0 [24.6–29.5]	26.0 [23.7–28.5]	24.8 [22.5–27.3	24.7 [22.3–27.1]	24.60 [22.3–27.1]	24.5 [22.2–27]	23.80 [21.5–26.2]	22.9 [20.6–25.3]
2 *Cuneatibacter caecimuris* DSM 29486^T^ (NZ_SGXF01000001.1)		100.00	25.7 [23.3–28.1]	23.0 [20.7–25.4]	25.8 [23.5–28.3]	19.2 [17–21.6]	19.0 [16.8–21.4]	25.4 [23.1–27.9]	23.1 [20.8–25.5]	30.1 [27.7–32.6]
3 *Eubacterium oxidoreducens* DSM 3217^T^ (NZ_FMXR01000031.1)			100.00	22.6 [20.3–25]	23.2 [20.9–25.7]	26.1 [23.8–28.6]	22.9 [20.6–25.3]	28.8 [26.4–31.3]	29.3 [26.9–31.8]	20.7 [18.4–23.1]
4 *Faecalicatena orotica* strain NLAE-zl-C242 (NZ_QGDL01000001.1)				100.00	22.0 [19.7–24.4]	21.6 [19.4–24.1]	21.7 [19.4–24.1	22.2 [20–24.7]	22.2 [19.9–24.6]	25.5 [23.2–28]
5 *Roseburia hominis* A2-183^T^ (NC_015977.1)					100.00	24.0 [21.7–26.5]	24.6 [22.3–27.1]	32.9 [30.5–35.4]	23.70 [21.4–26.1]	29.2 [26.9–31.7]
6 *Faecalicatena contorta* 2789STDY5834876 (NZ_CYZU01000001.1)						100.00	28.5 [26.2–31]	28.5 [26.1–31]	26.4 [24–28.8]	25.8 [23.5–28.3]
7 *Faecalicatena fissicatena* KCTC 15010 (NZ_LDAQ01000001.1)							100.00	26.4 [24.1–28.9]	25.3 [22.9–27.8]	23.1 [20.9–25.6]
8 *Anaerocolumna cellulosilytica* strain SN021^T^ (NZ_AP023367.1)								100.00	24.1 [21.8–26.5]	26.5 [24.1–29]
9 *Catonella morbi* ATCC 51271^T^ (NZ_KI535366.1)									100.00	21.0 [18.8–23.4]
10 *Herbinix luporum* strain SD1D^T^ (NZ_LN879430.1)										100.00

## Data Availability

The data presented in this study are contained within the article.
